# Detection of peritonsillar abscess using smartphone-based thermal imaging

**DOI:** 10.12669/pjms.332.12167

**Published:** 2017

**Authors:** Myung Jin Ban, Yunyoung Nam, Jae Hong Park

**Affiliations:** 1 Myung Jin Ban, Department of Medicine, The Graduate School of Yonsei University, Seoul, Korea Department of Otorhinolaryngology - Head and Neck Surgery, Soonchunhyang University College of Medicine, Cheonan, Korea; 2 Yunyoung Nam, Department of Computer Science and Engineering, Soonchunhyang University, Asan, Korea; 3 Jae Hong Park, Department of Otorhinolaryngology - Head and Neck Surgery, Soonchunhyang University College of Medicine, Cheonan, Korea

**Keywords:** Peritonsillar abscess, Thermography, Smartphone

## Abstract

**Objective:**

Smartphone-based thermal imaging was evaluated for its utility in the detection of peritonsillar abscesses.

**Methods:**

We describe six cases of peritonsillar abscess in which computed tomography and thermography scans of the neck were performed prior to surgery.

**Results:**

Open-mouth thermal photographic images were obtained preoperatively from patients, and asymmetric hot spots with significantly higher temperatures in the peritonsillar area were identified as abscesses.

**Conclusions:**

This new portable smartphone-based thermal imaging technique may be useful in the detection of peritonsillar abscesses.

## INTRODUCTION

Peritonsillar abscesses are among the most frequent and significant complications of acute tonsillitis. Immediate perioral incision and drainage may be critical to prevent deep neck infection in these patients. In cases with swelling of the neck or peritonsillar bulging in patients with tonsillitis, computed tomography (CT) scans of the neck are recommended to detect peritonsillar abscesses. However, some clinicians may hesitate to perform a CT scan because of the invasiveness of the radiation.

Recently, diagnostic infrared thermography has emerged as a medical imaging modality in the detection of various lesions. Thermography has the advantage of remote access with a non-contact, non-invasive technique. Thermal cameras are evolving with regards to speed, temperature, and spatial resolution, with a portable size and standardization protocol.^1^ No previous study has examined peritonsillar abscesses with thermography or the use of a portable camera for such detection. In this study, a commercially sold portable infrared camera attachable to a smartphone was used. Although the camera was designed for non-medical purposes, we evaluated its diagnostic performance in suspected cases of peritonsillar abscesses in patients with acute tonsillitis.

## METHODS

All the patients that visited our tertiary hospital clinic with symptoms of acute tonsillitis, such as a sore throat and fever, those with peritonsillar bulging and tender cervical lymphadenopathy were suspected to have unilateral peritonsillar abscesses. We used smartphone-based thermal imaging and CT scans to assess the suspicious peritonsillar and cervical lesions. We confirm that all procedures involved in this study comply with the ethical standards of the relevant national and institutional guidelines on human experimentation (Institutional Review Board of Soonchunhyang University Hospital Cheonan) and with the Helsinki Declaration of 1975, as revised in 2008. Written informed consent was obtained from all patients.

The following protocol was applied to reduce the performance bias of thermography. After the patient was made to relax for 30 minutes in a normothermic, humidity-controlled room, thermal images of bilateral tonsillar regions with temperature measurements were obtained by an independent examiner three separate times. The patient’s body temperature was also measured with a tympanic thermometer. To obtain the best image, the examiner tried to make the direction of the thermal camera coincide with that of the thermal radiation.

Patient exclusion criteria included the use of medications, cold packs, or bathing that affected body temperature within the six hour prior to thermal imaging, and excessive trismus. After the patient gargled with cold water (5°C), the dynamic thermal change was also evaluated over three minutes to compare both sides. The thermal imaging camera (Android Thermal Imaging Camera: Therm-App^®^, Opgal Optronic Industries Ltd., Israel, currently retails at under $1000) and corresponding free software via Google Play were used at a 10-cm distance from the mouth. The camera resolution was 384 x 288 pixels with a microbolometer imager (long wave infrared 7.5–14 μm), accuracy ±3°C or 3% (at 25°C), sensitivity < 0.07°C, and a temperature range of 5–90°C.

Patients with suspected peritonsillar abscesses were evaluated using CT scans. They were then treated with a perioral incision and drainage, resulting in a purulent discharge. Thermal images of the patients were reviewed retrospectively after drainage. The independent t-test was used to calculate the significance of the temperature difference between the peritonsillar abscess side and the tonsillitis side. A p value < 0.05 was considered significant. SPSS version 18.0 (SPSS, Chicago, IL) for Windows was used for the analysis. One month after discharge, thermal imaging was repeated to assess any remaining asymmetry.

## RESULTS

**Fig.1:**
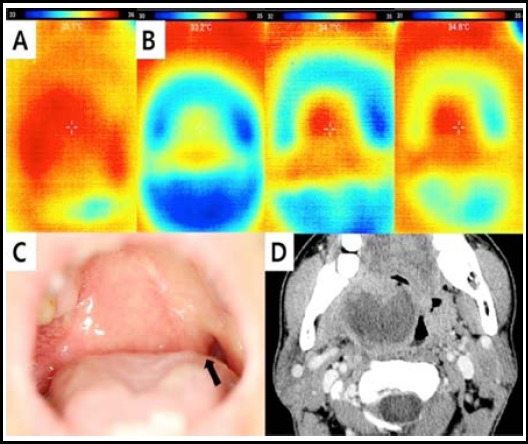
Smartphone-based thermal image of a peritonsillar abscess. Increase in temperature (35.1°C) of the right peritonsillar area, suspected to be caused by an abscess (A). Three serial dynamic images showing the change in temperature from 33.2°C to 34.8°C during the 3 min after gargling with cold water (5°C) over the right tonsillar area (B). The focal peritonsillar abscess is suspected via direct view due to the deviated uvula (black arrow) (C) and the CT scan confirms the suspected peritonsillar abscess (D). CT=computed tomography.

Thermal images (n=36) were reviewed in four women and two men (mean age, 48.8 ± 17.4 years and mean body temperature, 38.1 ± 0.33°C). Localized peritonsillar abscesses were detected by thermal imaging (Fig.1). These areas were detected at anatomical sites similar to those identified by the CT scans as rim-enhanced hypodense areas (mean size, 2.3 ± 0.19 cm). The difference in temperature was statistically significant (abscess side 35.4 ± 0.32°C, contralateral side 34.9 ± 0.37°C, mean ± standard deviation, p < 0.05). The mean difference was 0.48°C (± 0.12, standard error). After the peritonsillar abscesses were completely healed (1 month after treatment), thermal imaging from all patients revealed symmetrical sides.

## DISCUSSION

High-quality thermal cameras have beneficial clinical applications. With proper standardization, thermal cameras may be useful as a supplement for the diagnosis of breast cancer and inflammatory or vascular lesions.^1^ The highest specifications available for a commercial thermal imaging camera are 1280 x 1024 pixels, in terms of resolution, and ±1°C or ±1%, in terms of reading accuracy. Otolaryngological thermography has been applied in cases of sinusitis, stomatitis, gastroesophageal reflux disease, Frey’s syndrome, free flap monitoring, and monitoring after third molar surgery.^2^–^6^ No study has been conducted using thermal imaging for the detection of peritonsillar abscesses.

Since many factors affect thermal emission and detection, previous studies have analyzed the asymmetry assessed by our study.^2^,^4^ The known mean temperature difference between the two sides of various body segments is 0.11–0.5°C, and our results showed a similar difference, despite detection of pathologic inflammatory lesions.^7^ Deng et al. reported that water and a 75% medical ethanol solution sprayed on the skin can significantly enhance the sensitivity of temperature mapping and improve the diagnostic accuracy during the early stages of deeply embedded tumors.^8^ To improve the contrast of the peritonsillar abscesses, we also used cold water (5°C) gargling, and an improvement in diagnostic temperature mapping was observed (Fig. 1B). Although the number of patients was small and the resolution low, there was a clear thermographic difference in the location of the peritonsillar abscesses.

This retrospective case study failed to verify the diagnostic value of thermography for detecting peritonsillar abscesses, and therefore, further clinical trials are warranted. To verify the diagnostic value, thermal images should be obtained prospectively from patients with a simple sore throat, and compared to images obtained using computed tomography (CT) scanning, the gold standard.

Although a physical examination and CT scan are sufficient for otolaryngologists to diagnose a peritonsillar abscess, we expect the easy and applicable smartphone-based thermal camera to be a potential diagnostic tool for general health care of patients presenting with sore throats.

## CONCLUSION

Non-invasive medical infrared thermography has evolved to show various applications. A portable smartphone-based thermal camera may enable detection of the peritonsillar abscesses in patients with tonsillitis.
